# A novel dual-layer approach towards omniphobic polyurethane coatings†

**DOI:** 10.1039/c9ra04923a

**Published:** 2019-08-27

**Authors:** Fahad Khan, Ajmir Khan, Mohammad O. Tuhin, Muhammad Rabnawaz, Zhao Li, Muhammad Naveed

**Affiliations:** School of Packaging, Michigan State University 448 Wilson Road, East Lansing Michigan 48824-1223 USA rabnawaz@msu.edu +1-517-432-4870; Department of Chemistry, Hazara University Mansehra Khyber Pakhtunkhwa 21300 Pakistan

## Abstract

Omniphobic surfaces have a plethora of applications ranging from household paints to sensors. The predominant practice of fabricating those materials/surfaces is to use fluorinated materials which are environmentally harmful, and thus have limited practical applications. In this study, we report a novel dual-layer approach of fabrication towards omniphobic surfaces using polyurethane (PU) as a matrix and polydimethylsiloxane (PDMS) as a self-cleaning ingredient. This approach was also used to produce omniphobic PU nanocomposites, where nanofillers (*e.g.*, nanoclay, cellulose nanocrystals (CNCs) and graphene oxide (GO)) were incorporated. The resultant coatings were investigated for their performance, such as optical clarity, durability, and self-cleaning properties. In addition, scanning electron microscopy (SEM) was used for microstructural analysis of the obtained coatings. The facile nature of fabrication and the use of PDMS, an environmentally benign material relative to fluorinated chemicals, thus offer an eco-friendly sustainable scheme for practical applications aimed at omniphobic purposes.

## Abstract 2

Omniphobic surfaces have a plethora of applications ranging from household paints to sensors.

## Graphical abstract

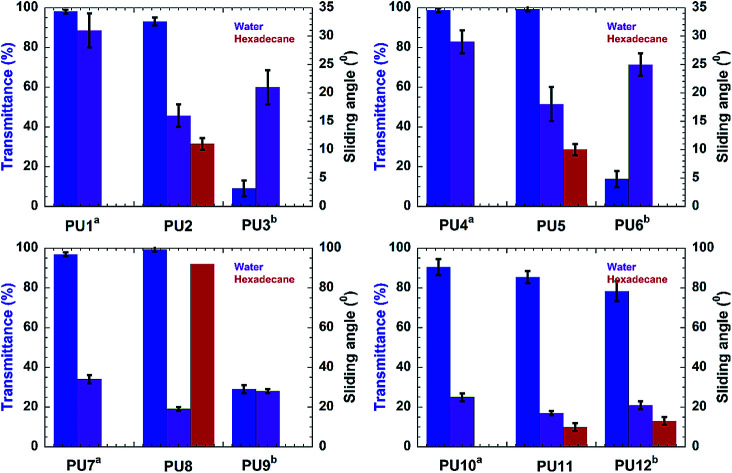

## Introduction

Durable and optically clear omniphobic coatings repelling both polar and non-polar liquids are highly desirable for a plethora of applications (*e.g.*, household, sensors, fuel transport, solar panels, *etc.*).^1,2^ To fabricate omniphobic surfaces, researchers often use lotus leaf^3,4^ and pitcher plant^2^ models. The lotus leaf model inspires the creation of omniphobic surfaces with a very rough architecture chemically modified with low surface energy materials and polymers.^4,5^ These surfaces are characterized by very high static contact angles (>150°) for both polar and non-polar liquids. For example, in an elegant study, Deng *et al.*^6^ utilized candle soot in a coating matrix to replicate the lotus leaf model that yielded durable omniphobic coatings. Recently, Pan *et al.*^7^ reported the use of cyanoacrylate and fluorosilane to prepare durable surfaces with excellent omniphobicity. However, the real-life applications of rough omniphobic surfaces are hindered because of their failure at high pressure caused by the entry of liquids into voids of a rough surface,^8^ and poor abrasion resistance.^9^ A further concern arises by the use of PFAS and other fluorinated materials that are environmentally persistent as well as harmful.^10^ In addition, with rare exceptions, rough omniphobic surfaces are optically translucent or opaque that limits their applications where optical clarity is desirable.^11,12^ Therefore, smooth omniphobic surfaces have recently attracted significant attention.^2,13,14^

Smooth omniphobic surfaces, characterized by low static contact angles, have very low sliding angles for both polar and non-polar liquids. Virtually all types of liquids readily slide off smooth-omniphobic surfaces because of the dewetting properties imparted by the conformational changes of the low surface energy materials, often low *T*_g_ fluorinated polymers, on their surface.^13–15^ For example, in a pioneering work, slippery liquid-infused porous surfaces' (SLIPSs),^2^ where a fluorinated oil was infused into micro/nanoporous surfaces, that imparted excellent omniphobic properties. However, SLIPSs are often plagued with poor clarity, and tedious surface modification before fabrications. In addition, SLIPSs approach is still less suitable where the surfaces are encountering mechanical rubbing as the infused liquid can be rubbed off the SLIPS; irrespective of some reported improvements^16,17^ of robustness in silicon oil-infused surfaces. The constructing self-restoring surfaces was also found to be useful as an innovative approach of compensation for such scrubbed off SLIPS.^18^ Another commonly used approach to fabricate smooth surfaces utilizes chemical grafting of a thin layer of low surface energy polymers such as poly(hexafluoroisopropylene oxide) has also been successfully used to obtain omniphobic surfaces.^19^ However, the resultant thin coatings have inferior mechanical properties, in addition to their reliance on the use of expensive and environmentally unfriendly fluorinated materials. The layer-by-layer method can also yield smooth self-cleaning coatings with accurate thickness,^20–22^ which, as a downside, requires tedious fabrication process making them less viable for real-life applications.

To address the durability, conventional coating formulations (*e.g.*, epoxies, urethanes) are sometimes loaded with omniphobic polymers.^23,24^ However, these omniphobic coatings are rarely clear because of the phase separation of the constituting components in conventional coatings.^24,25^ In recent efforts, in this regard, polyol-*graft*-Krytox- and polyol-*graft*-PDMS-copolymers were incorporated into PU coating to improve the clarity, besides bringing in excellent water and oil repellency.^26–28^ The presence of polyol-*graft*-Krytox- and polyol-*graft*-PDMS enhanced the compatibility between low surface energy PDMS (or Krytox) and the polar PU matrix, and as a result of that, the formation of clear durable coatings was enabled. Nevertheless, the graft copolymers prepared in this study dependent on complicated synthetic processes as well as the use of environmentally harmful chemicals and solvents.

Hydrophilic nanofillers such as nanoclays, graphene oxide (GO), cellulose nanocrystals (CNCs), carbon nanotubes (CNTs), and pigments are often added to improve certain properties.^29–33^ A common problem with hydrophilic nanofillers could be their poor dispersability in the presence of low surface energy polymers/chemicals (*e.g.*, PDMS, fluorochemicals). However, due to their hydrophilic nature, such composites lose their performance as a result of the absorption of moisture from air.^34–37^ To address this problem of these composites, hydrophobic nanofillers have been attempted.^38^ Nevertheless, hydrophobic nanofillers have poor dispersibility in a relatively hydrophilic matrix; and thus was found to result in flocculation of nanofillers in the final coatings.^39,40^ Because of these reasons, preparation of nanocomposites with concurrent omniphobic properties as well as highly dispersed nanofillers is a challenging task.

Herein we report a facile approach for the fabrication of durable and optically clear omniphobic urethane coatings and their nanocomposites using PDMS instead of fluorochemicals. This approach utilizes commercially available ingredients (polyol, polyisocyanate, PDMS–NH_2_) without any modification, instead of using tedious grafting chemistry. The resultant coatings were tested for water-, oil- and ink-repellency as well as their properties related to optical transparency. The methods developed in this study is highly versatile as demonstrated by the fabrication of clear and durable omniphobic PU coatings as well as their composites with nanoclay, CNC, and GO.

## Results and discussion

**Scheme 1:**
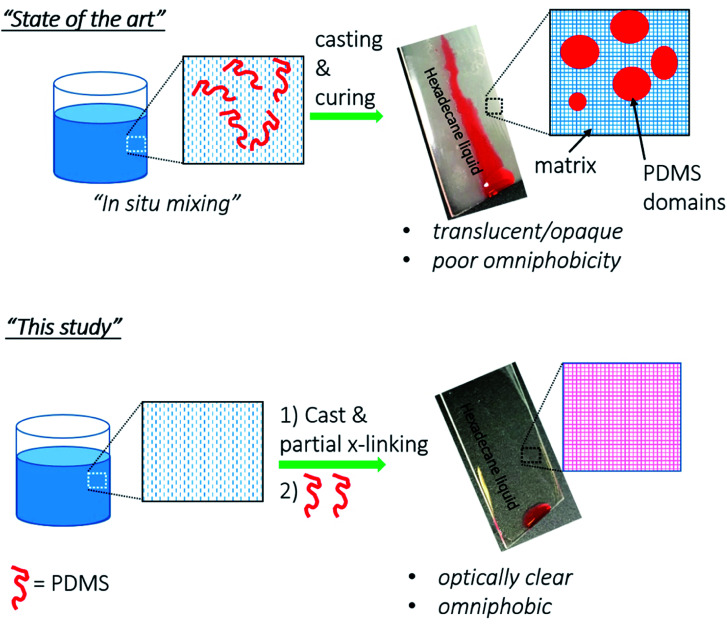
Schematic illustrations of our novel top-layer approach (“This study”) *versus* the conventional approaches (“State of the art”) to fabricate omniphobic surfaces.

Our approach towards omniphobic PU coatings are based on the use of low surface energy PDMS (surface energy = 20 mN m^−1^).^35^ However, others and we observed that PDMS, if mixed with urethane formulation, undergo phase separation in urethane matrix due to the incompatibility of non-polar PDMS and polar urethane matrix. This phase separation is illustrated in Scheme 1 as “State of the art”.^23,27,41–43^ Due to the phase separation, PDMS chains aggregate in large domains, and thus scatter visible light resulting in hazy/translucent films.

To address the phase separation of PDMS in the urethane, we envisioned to apply PDMS on a semi-crosslinked PU coating (see Scheme 1 “This study”). According to our strategy, once PU coating is vitrified (partially crosslinked) on a substrate, then, PDMS–NH_2_ solution will be applied as a top-layer onto the partially crosslinked PU. Partial crosslinking was selected to facilitate PDMS solution permeation into the PU matrix from the top. To avoid phase separation, PDMS–NH_2_ was chosen because the NH_2_ group of the PDMS–NH_2_ reacts efficiently with NCO of the partially crosslinked PU, and thus PDMS will covalently bond to the PU matrix. PDMS, applied on top, is expected to be distributed into bulk portions, and such diffusion will be enabled due to the presence of partially crosslinked PU matrix. The final obtained films will be single coating having incorporated PDMS chains dispersed throughout the matrix; but highly enriched in the top layers where they are needed the most for anti-smudge properties.

**Scheme 2:**
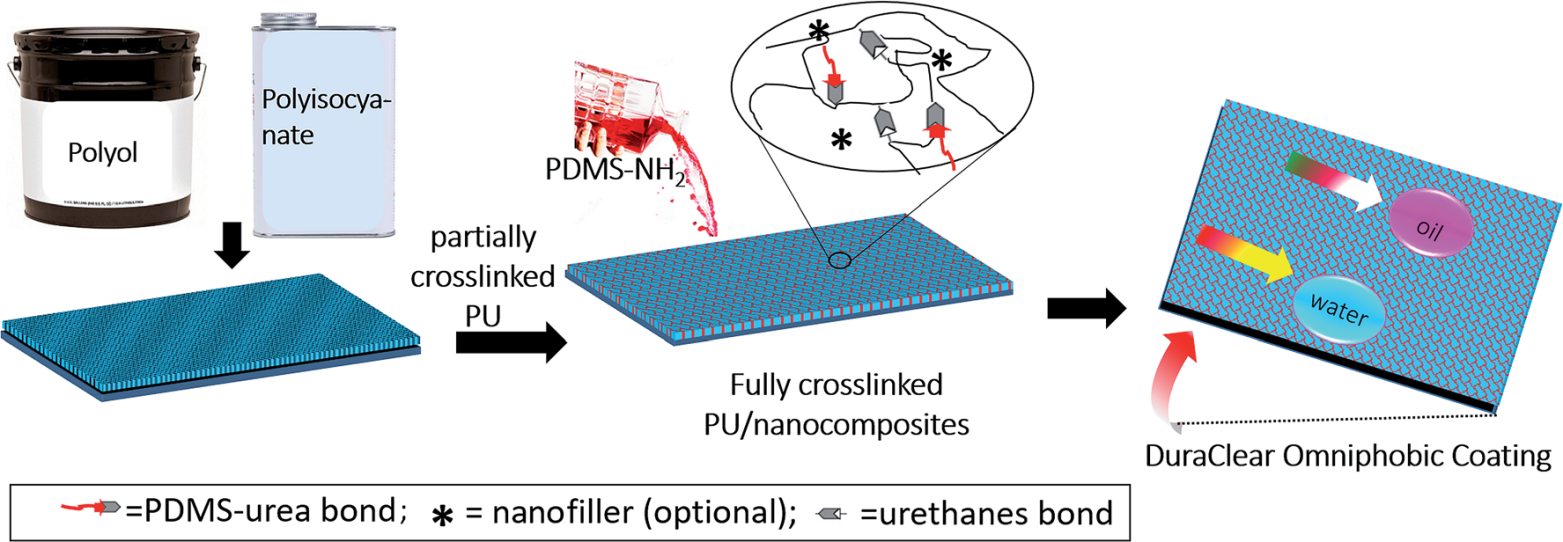
Schematic illustration of the omniphobic PU coatings preparation using our novel top-layer approach.

To prove the above hypothesis, various PU-based coatings were prepared by the process illustrated in Scheme 2. PU coatings were prepared using a polyol (the ^1^H NMR spectrum and chemical structure are shown in Fig. S1†) and a hexamethylene diisocyanate trimer (HDIT, the ^1^H NMR spectrum and chemical structure are shown in Fig. S2†). Polyols and HDIT were mixed in 1 : 1.1 equivalent ratios. In certain coatings, nanofillers were also added to the PU formulations. Once the polyol and isocyanate mixtures were applied on a glass substrate, PU coating was allowed to vitrify by applying a short period of thermal treatment. PDMS–NH_2_ solution was then applied on top of the vitrified coating *via* a drop cast method, which was followed by full curing at 120 °C. ATR-FTIR analysis was used to monitor the curing of urethane formulations at various time intervals. After 360 min, complete curing of the PU was achieved, as evident by the disappearance of peak at 2270 cm^−1^ corresponding to the NCO groups of the urethane (Fig. S3†). Therefore, all urethane coatings investigated in this study were cured at 120 °C for 360 min prior to further analysis.

To investigate the importance of the partial crosslinking necessary for this dual-layer approach before PDMS treatment, fully cured PU were prepared and treated with PDMS–NH_2_. Fig. S4† shows the FTIR analysis of the completely cured urethane as suggested by the absence of NCO peak at 2270 cm^−1^. The resultant coating obtained after the dip-coating of the cured PU from PDMS–NH_2_ followed by the subsequent washing with hexane did not show anti-ink (Fig. S5†) and self-cleaning properties (Fig. S6†). Thus, partial crosslinking is critically important to impart self-cleaning properties in this dual layer approach.

In this study, four sets of PU coatings were prepared with the compositions listed in Table S1.† These included Urethane without nanofillers (PU1-3), urethane/nanoclay (PU4-6), urethane/CNC, (PU7-9), and urethane/GO (PU9-12). The performance of each of these four sets of coatings were tested against their respective controls. One control was the system lacking PDMS, while the other control was prepared *via* direct mixing of PDMS–NH_2_ in the coating formulation referred as “*in situ*” mixing in this article.

**Fig. 1:**
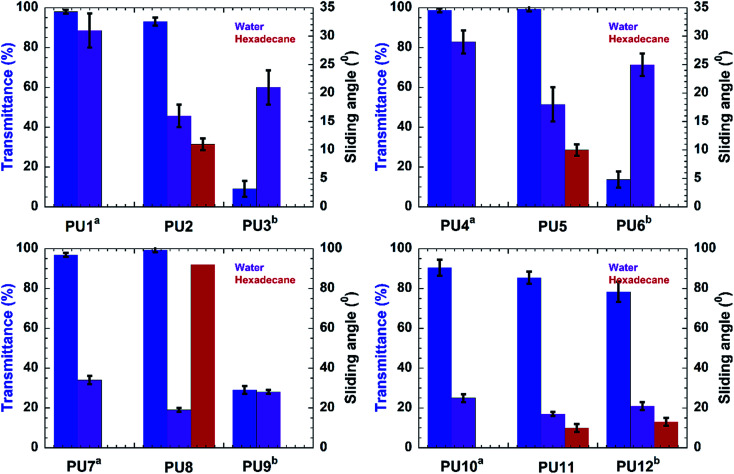
Transmittance with respect to the visible light and sliding angle corresponding to different compositions of the coating materials. On the axes of sliding angle data, the purple bars represent the sliding angle of water while the red bars represent the sliding angle of hexadecane. The absence of sliding angle data for any coated material indicates the wetting of the surface with hexadecane. The superscript notations indicate the variation in composition and the method of preparation: a = no PDMS–NH_2_ was used; b = prepared *via* “*in situ*” mixing.

**Fig. 2:**
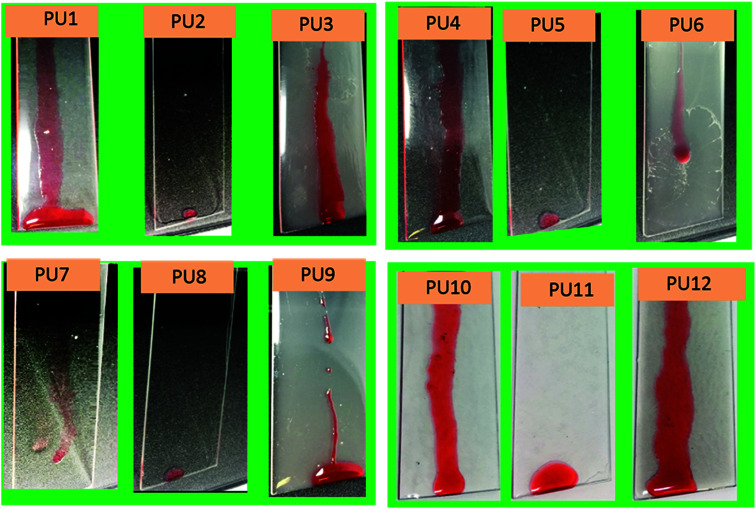
Anti-smudge properties of various PU samples. Hexadecane droplets sliding behaviors on PU1-12 samples.

Samples PU1-3 corresponds to PU coatings without any nanofiller. The PU2 coating, prepared by top-layer approach, exhibited excellent water and oil-repellency relative to the *in situ*-prepared analog, PU3. For example, PU2 showed water and hexadecane sliding angles of 16 ± 2° and 11 ± 1°, respectively, for the top-layer approach. Also, sample PU2 showed better optical clarity (93 ± 2 %*T*) than that observed for the translucent PU3 (9.1 ± 4 %*T*) (Fig. 1). The superior optical properties exhibited by the PU2 (see Fig. 2) coating obtained *via* top-layer approach can be attributed to the absence of phase separation of the PDMS in urethane matrix. For the “*in situ*” system, PDMS underwent phase-separation in urethane matrix due to the incompatibility between hydrophilic polyol–HDIT matrix and hydrophobic PDMS chains, as is evident from the poor clarity of PU3 samples.

Encouraged by the excellent performance offered by the top-layer approach for omniphobic PU coating, we investigated the effectiveness of this approach for fabrication of PU/nanoclay, PU/CNC and PU/GO coatings (see Table S1†). Nanofillers were dispersed in the PU formulation *via* sonication. Among the PU/Nanoclay composites, only that prepared by the top-layer approach (sample PU5, Table S1†) showed good omniphobic performance. For example, the PU5 exhibited sliding angles of 18 ± 3° and 10 ± 1° for water and hexadecane, respectively. Meanwhile, the “*in situ*” method yielded coating (sample PU6, Table S1†) did not exhibit hexadecane repellency. The hexadecane sliding behavior of all samples is shown in Fig. 2. Also, the PU5 sample prepared *via* top-layer approach had good optical clarity (99.2 ± 1 %*T*) (Fig. 1) in contrast to the optically translucent PU6 sample (13.7 ± 4 %*T*) (Fig. 1) prepared *via* “*in situ*” technique.

PU/CNC (samples PU7-9, Table S1†) and PU/GO (samples PU10-12, Table S1†) nanocomposites were prepared using the “*in situ*” method as well as our novel top-layer approach. Samples PU8 and PU11, which were prepared by the top-layer approach, offered superior optical clarity as well as water- and oil-repellency (Fig. 1). For example, sample PU8 had better optical clarity (99.2 ± 1 %T) than it's “*in situ*” derived counterpart, PU9 (29 ± 2 %*T*). Likewise, sample PU11 had better optical clarity than the corresponding “*in situ*” – fabricated sample, PU12 (Fig. 1).

**Fig. 3:**
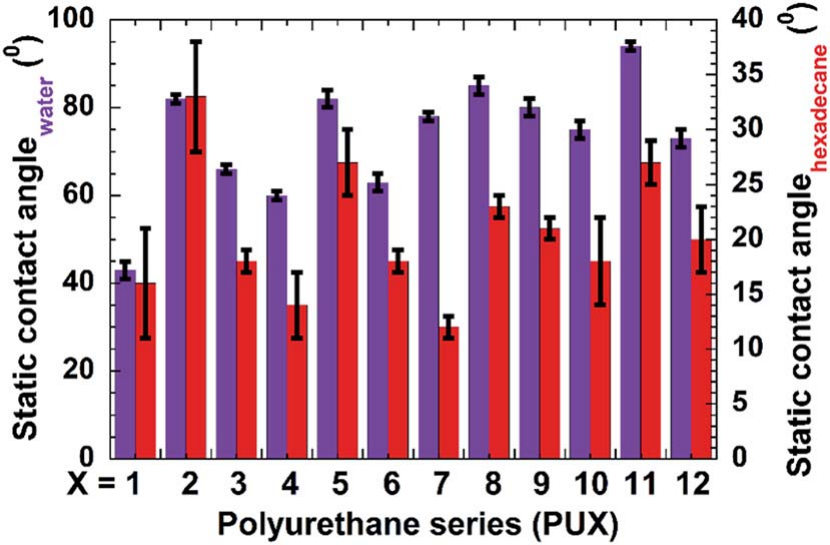
Static contact angle corresponding to samples PU1-12. Here, the X denotes the number designated in nomenclature of the samples. For example, the data corresponding to 1 on *x*-axis are representative data for PU1.

We also recorded static contact angles for all samples used in this study (Fig. 3). As expected, the incorporation of PDMS increased the static contact angles with respect to all test liquids. The observed contact angles were also highly dependent on the type of nanofillers used. The PU samples prepared by top-layer approach (PU-2, -5, -8, -11) showed higher static contact angles in case of both water and hexadecane droplets, which further validated the effectiveness of our novel top-layer approach.

**Fig. 4:**
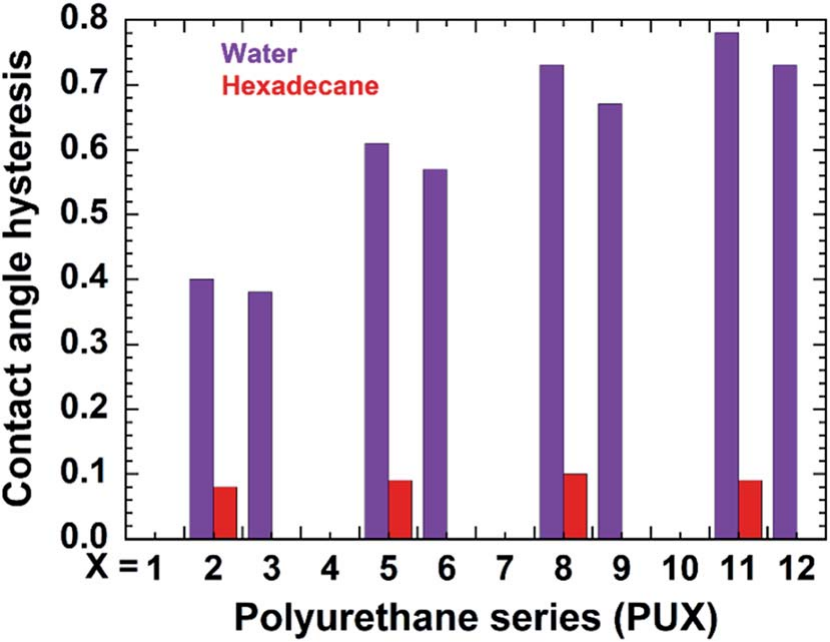
Static contact angle hysteresis for water and hexadecane on the PU1-12 coatings. The naming style of the PU-series was followed as it was mentioned in case of Fig. 3. The absence of bar for any PUX sample indicates that liquid wets the corresponding surface.

The contact angle hysteresis of samples PU1-12 were also determined for water and hexadecane (Fig. 4). Samples lacking PDMS (PU1, PU4, PU7, and PU10) showed no pinning of the water on their surface. While samples having PDMS either prepared by top-layer or *in situ* approach showed contact angle hysteresis in the range of 0.38–0.78. For PU2 and PU3 having incorporated nanofiller showed low hysteresis of ∼0.4. Interestingly, low hexadecane hysteresis were obtained for all samples prepared by top-layer approach, while the *in situ* samples, as well as the samples lacking PDMS were wet by hexadecane.

**Fig. 5:**
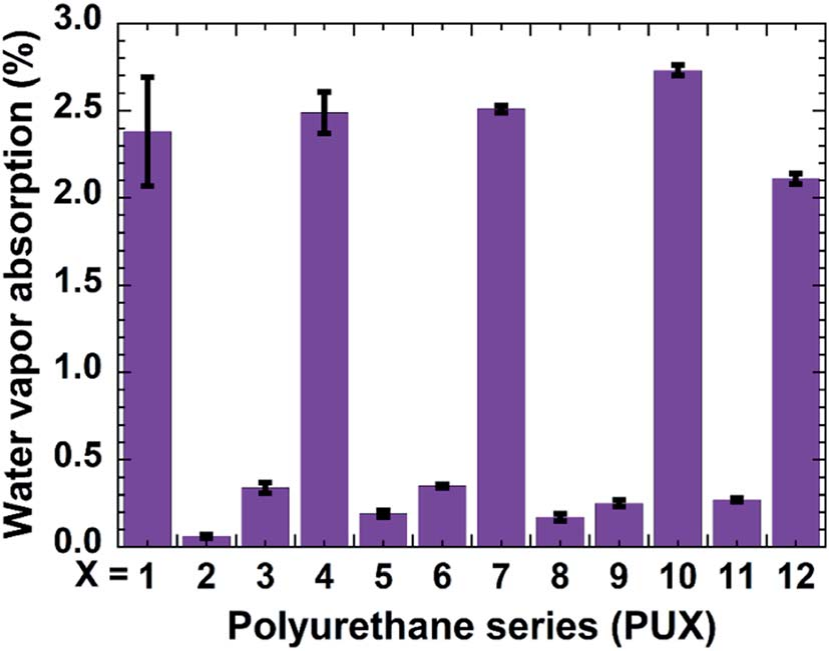
Water vapor absorption of PU1-12 coatings.

Aside from exhibiting good omniphobicity and optical clarity, our coatings also showed excellent resistance against humidity. A coating, upon the absorbance of a significant amount of water vapor (moisture), loses their adhesion performance, become vulnerable to delamination and turns mechanically weak. Bearing this practical consideration in mind, we investigated the water vapor absorption of the PU1-12 samples (Fig. 5).

Samples lacking PDMS typically showed high water vapor absorption. However, as anticipated, the addition of PDMS *via* the “*in situ*” approach reduced the water absorption of resultant coatings due to the water-repellent nature of PDMS. More importantly, a remarkable reduction in water vapor absorption was observed for the films prepared *via* the top-layer approach. For example, the water vapor absorption was decreased by 97.5% for the PU2 coating developed by the top-layer procedure relative to the PDMS-free PU1 coating. In the case of nanoclay-, CNC-, and GO-containing composites, the respective water absorption values exhibited by the top-layer-derived coatings were also reduced by approximately 92%, 90%, and 90%, respectively, relative to their PDMS-free counterparts (see Fig. 5). Thus, the coatings prepared *via* top-layer approach showed excellent barrier characteristics against moisture absorbance. The superior water vapor barrier properties for PU samples carrying PDMS correspond to the better water resistance and the resultant anticipated reduced swelling of the coating by the water vapor relative to the PU without PDMS. PU-2 showed better performance relative to PU-5, PU-8, and PU-11, possibly due to the very hydrophilic nature of the nanofillers causing more water vapor absorption than neat urethane. The water vapor barrier properties are in corroboration with the better water contact angle hysteresis of the PU-2 compare to PU-5, PU-8, and PU-11 (see Fig. 4). Detail investigation of the PU and nanofillers systems will be explored in the future.

**Fig. 6:**
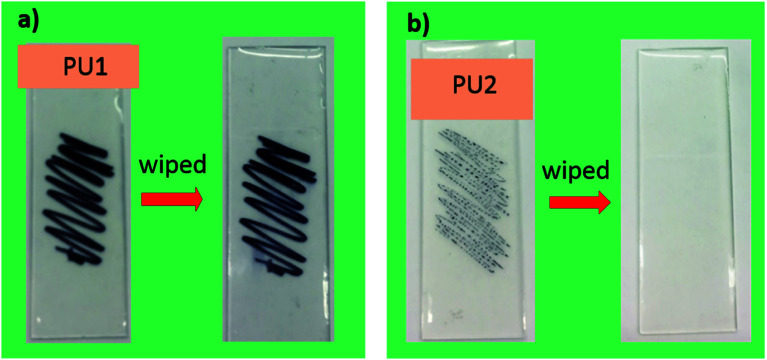
Ink traces left behind by a permanent ink marker on PU1- and PU2-coated glass plates. Photos were taken before and after the samples had been subjected to wiping treatment are also shown.

Fig. 6 depicts the response of PU1 and PU2 against permanent sharpie marker. As can be seen, PU1 not only receives ink with ease but also ink cannot be erased from their surface. In contrast, PU2 showed good ink-resistance. For example, ink shrank on their surfaces, as well as permanent ink was easily wiped away with a Kimwipe as shown in Fig. 6b, indicating excellent anti-graffiti nature of the PU2 samples.

Tensile tests were also used to determine the mechanical properties of omniphobic PU coatings. One would expect an increase in tensile strain, decrease in tensile stress as well as Young's modulus for the PU containing PDMS relative to reference PU due to the addition of more elastic PDMS into the matrix. However, we are using very little amount of the PDMS in the PU matrix, therefore, no significant change was anticipated in the tensile properties (Table 1). Overall, Young's moduli remained essentially unchanged except for PU8. Tensile strain increased for the PU2 with respect to PU1 because of the addition of PDMS, while tensile stress did not change significantly except for PU8 and PU11. Considering the excellent tensile properties of tested samples, this novel top-layer approach can be used to prepare omniphobic films with excellent mechanical durability.

**Table 1:** Mechanical properties of the selected urethane coatings

Sample#	Strain (%)	Stress (MPa)	Young's modulus (MPa)
PU1	7.8 ± 2.3	47 ± 8	1402 ± 24
PU2	8.4 ± 0.4	49 ± 6	1368 ± 51
PU7	7.2 ± 1.5	54 ± 9	1813 ± 51
PU8	5.8 ± 0.6	35 ± 13	1135 ± 241
PU10	8.5 ± 0.4	31 ± 10	1327 ± 130
PU11	5.4 ± 0.7	40 ± 6	1308 ± 157

**Fig. 7:**
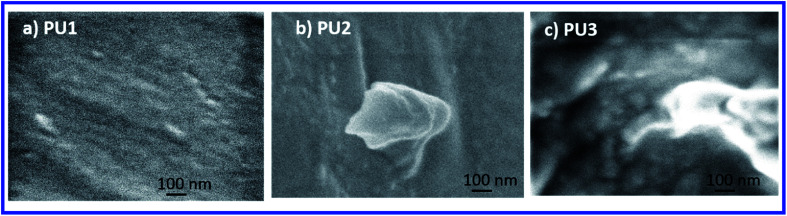
SEM images of (a) PU1; (b) PU2; and (c) PU3.

SEM images of the PU coatings (PU1, PU2, and PU3) are shown in Fig. 7. As shown in Fig. 7a and b, the coatings are relatively smooth with no visible evidence of phase separation. In contrast, PU3 shows the non-uniform distribution of phase-separated PDMS as is demonstrated by the brighter domains in the SEM image. Also, the phase-separated PDMS domains, of various shapes and of various sizes ranging from ∼50 nm to several microns in diameter, were possibly caused by the aggregation of small phase-separated domains into larger ones. Thus, SEM characterization confirmed that the opaque PU3 films prepared *via* the “*in situ*” approach had indeed undergone phase segregation. Besides, AFM analysis were performed to understand the surface texture for selected samples. As shown in Table S2,† PU1, PU2, PU3, PU5, and PU11 coatings had smooth surfaces with root means square below 1 nm.

## Conclusions

In this study, we have developed an environmentally friendly novel approach to fabricate omniphobic PU and PU/nanocomposites coatings that are optically clear and mechanically durable, in addition to possessing excellent water-, oil- and ink-repellency. Besides, the prepared coatings showed a remarkable reduction in the water vapor absorption. SEM analysis confirmed the absence of phase separation of the PDMS in the PU matrix prepared by the top-layer approach. Considering the fluorine-free nature as well as the use of commercial ingredients, along with the characteristic properties of resultant coatings, this facile approach will benefit various areas of basic and applied sciences. Therefore, this study embodies a simplistic, yet practically applicable method and materials with wide window of potential exploration and tunability in the field of omniphobic materials. We are currently working on the additional aspects (*e.g.*, PDMS distribution in the matrix, weather-resistance and formulations to further increase the omniphobic performance) of this novel dual-layer method.

## Experimental

### Materials

Acetone (Fischer scientific, 99.7%), PDMS–NH_2_ (monoaminopropyl terminated polydimethylsiloxane, *M*_n_ = 2000 g mol^−1^, GELEST, Inc.), hexadecane (Sigma Aldrich, 99%), cooking oil (local Meijer store, Canola oil), montmorillonite clay (Sigma, 99%) were used as received. Graphene oxide (Sigma Aldrich, dispersed in water) was dialyzed against acetone to remove water prior to use. Propylene oxide-based triol (polyol) and hexamethylene diisocyanate trimer (HDIT) were generously donated by Covestro and were characterized before ^1^H NMR.

### Methods

#### Preparation of urethane and urethane/nanofiller coatings without PDMS

Polyol (P1, 0.70 mL, 2.4 mmol) was dissolved in acetone (1.0 mL) and then HDIT (1.1 mL, 2.5 mmol) was added to this solution. The mixture was then sonicated at room temperature for 1 h. Subsequently, 0.7 mL of this solution was cast onto a glass slide (2.54 cm × 7.62 cm), which was then left at room temperature until the solvent had evaporated. After complete evaporation of the solvent, the sample was cured at 120 °C for 6 h. The urethane coating had a thickness of ∼267 ± 8 μm.

During the preparation of urethane/nanofiller films (without PDMS), a polyol (P1, 0.70 mL, 2.4 mmol) was initially dissolved in acetone (1.0 mL). The nanofillers (nanoclay, GO or CNC, 4 mg dispersed in 0.2 mL of acetone) were subsequently added to this solution and sonicated for 20 min at room temperature. HDIT (1.1 mL, 2.5 mmol) was added to this solution and stirred with a vortex mixer for ∼1 min and then sonicated at room temperature for 1 h. Subsequently, 0.7 mL of this solution was cast onto a glass slide (2.54 cm × 7.62 cm) and left at room temperature until the solvent had evaporated. After complete evaporation of the solvent, the sample was cured at 120 °C for 6 h. The urethane coating had a thickness of ∼267 ± 8 μm.

#### Preparation of omniphobic urethane and urethane/nanofillers coating *via* the top-layer approach

The polyol (P1, 0.70 mL, 2.4 mmol) was dissolved in acetone (1.0 mL) and then HDIT (1.1 mL, 2.5 mmol) was added. The mixture was sonicated at room temperature for 1 h and 0.7 mL of this solution was cast onto a glass slide of (2.54 cm × 7.62 cm). After solvent evaporation, the coatings were placed in an oven at 120 °C for 5 min to trigger partial crosslinking. The coatings were then cooled to room temperature. PDMS–NH_2_ (*M*_n_ = 2000 g mol^−1^, 4.9 mg dissolved in 0.6 mL of acetone) was added onto the top-layer using a syringe. The sample was left to allow for solvent evaporation to occur at ambient conditions, and subsequently cured at 120 °C for 6 h. The final coating had a thickness of ∼267 ± 8 μm.

The following top-layer approach was used to obtain omniphobic urethane/nanofiller coatings. First, the polyol (P1, 0.70 mL, 2.4 mmol) was dissolved in acetone (1.0 mL). A nanofiller (nanoclay, GO or CNC 4.0 mg dispersed in 0.2 mL of acetone) was then added to this solution and sonicated for 20 min at room temperature. Subsequently, HDIT (1.1 mL, 2.5 mmol) was added to this solution and vortexed for ∼1 min before it was sonicated at room temperature for 1 h. A 0.7 mL portion of the resulting solution was then cast onto a glass slide with dimensions of 1 × 3, and left to allow solvent evaporation to occur under ambient conditions. After solvent evaporation, the coatings were placed in an oven at 120 °C for 5 min to trigger partial crosslinking. The coatings were then cooled to room temperature. PDMS–NH_2_ (*M*_n_ = 2000 g mol^−1^, 4.9 mg dissolved in 0.6 mL of acetone, 0.0025 mmol of NH_2_) was added on the top-layer using syringe. The sample was left to allow solvent evaporation to proceed under ambient conditions, and then cured at 120 °C for 6 h. The final coating had a thickness of ∼267 ± 8 μm.

#### Preparation of urethane and urethane/nanofiller coatings *via* the “*in situ*” mixing of PDMS

The polyol (P1, 0.70 mL, 2.4 mmol) was dissolved in acetone (1.0 mL) and HDIT (1.1 mL, 2.5 mmol) was then added prior to the addition of PDMS–NH_2_ (*M*_n_ = 2000 g mol^−1^, 4.9 mg dissolved in 0.2 mL of acetone, 0.0025 mmol of NH_2_). This mixture was subsequently sonicated at room temperature for 1 h, and then 0.6 mL of the resulting solution was cast onto a glass slide, and left to allow solvent evaporation to occur. This sample was subsequently cured at 120 °C for 6 h in an oven.

In the case of urethane/nanofiller coatings prepared *via* “*in situ*” PDMS mixing, the polyol (P1, 0.70 mL, 2.4 mmol) was first dissolved in acetone (1.0 mL) and, then, HDIT (1.1 mL, 2.5 mmol) was added afterwards. A nanofiller (nanoclay, GO or CNC, 4 mg dispersed in 0.2 mL of acetone) was subsequently added to this solution followed by sonication for 20 min at room temperature. This was followed by the addition of PDMS–NH_2_ (*M*_n_ = 2000 g mol^−1^, 4.9 mg dissolved in 0.2 mL of acetone, 0.0025 mmol of NH_2_) and sonication of the resultant solution at room temperature for 1 h. Subsequently, 0.6 mL of the resulting solution was cast onto a glass slide and the solvent was allowed to evaporate at room temperature prior to curing at 120 °C for 6 h in oven.

#### Sliding angle measurements

The sliding angles of the coatings were measured with a coefficient of friction tester (MTS Tensile Testing Machine Type T5001) using deionized water (droplet volume of 75 μL), and hexadecane (droplet volume of 10 μL) as test liquids at room temperature.

#### Optical transmittance measurements

The optical transmittance of the coating samples was recorded in the range of 190–800 nm using a PerkinElmer Lambda 25 UV-vis spectrometer. The values reported in the article correspond to the transmittance (%*T*) at 540 nm where an uncoated glass slide was used as a reference.

#### ATR-FTIR analysis

ATR-IR analysis of the urethane coatings was performed using a Schimadzu IR Affinity-1S spectrometer equipped with a diamond crystal stage. Urethane coatings were cured for various durations at 120 °C, and the films were scratched from their glass substrates and placed on the diamond crystal stage and 64 scans were run for each sample.

#### Anti-graffiti tests

Ink was applied onto selected coatings *via* a Sharpie® permanent marker. The anti-graffiti performance of the coating samples was evaluated by visual observation of the ink that was transferred onto different coating samples as well as the ink left behind after wiping with a Kimwipe tissue.

#### Water gain analysis of the PU coatings

PU1-12 samples were prepared in aluminum baking pan. Prior to the water absorption testing, samples were first conditioned in an oven at 120 °C for 1 h without detaching it from aluminum cup. These samples were then placed in humidity chamber at 85% relative humidity and at 37 °C for different timespans, including 1, 2, 3, 4 and 24 h. The weight gain due to water vapor absorption was recorded using a microbalance.

#### SEM analysis of PU coatings

Scanning electron microscopy (SEM) images were recorded using a JEOL 7500F system that was equipped with a cold field emission emitter. The samples were cut with the aid of a sharp surgical blade and then, with help of EP resin, were adhered to a disc and kept under vacuum overnight. These samples were subsequently coated with Osmium *via* aero-spray method prior to imaging.

#### Mechanical properties of the PU coatings

The tensile properties were evaluated on films cut into 2.5 cm × 0.6 cm sections and were tested using an Instron 5565P6021 testing system following ASTM D882.

## Funding information

The authors declare no funding for this work.

## Author contributions

MR conceived the idea. MR and FK designed the experiments, while FK run most of the experiments. ZL helped in the characterization of the samples as well as manuscript write up. MT helped with characterization, and analysis. MN help to create make up data for the revisions. MR is thankful to TSGTD-MSU for the partial support of this research work.

## Conflicts of interest

The authors declare no competing interests.

## Supplementary Material

RA-009-C9RA04923A-s001
