# Supplementary material for: A novel dual-layer approach towards omniphobic polyurethane coatings
Source: RSC Adv. 2019 Aug 27;9(46):26703–11. doi: 10.1039/c9ra04923a (PMC9070450; doi:10.1039/c9ra04923a)
Supplement: RA-009-C9RA04923A-s001 [file RA-009-C9RA04923A-s001.pdf]

Electronic Supplementary Material (ESI) for RSC Advances.
This journal is © The Royal Society of Chemistry 2019

SUPPORTING INFORMATION

Novel Dual-Layer Approach towards Omniphobic Polyurethane Coatings

Fahad Khan[a,b], Ajmir Khan[a], Mohammad O. Tuhin[a], Muhammad Rabnawaz[a,*], Zhao Li[a,] Muhammad Naveed[a]

[a]School of Packaging, Michigan State University, 448 Wilson Road, East Lansing, Michigan 48824-1223, United States of America.

[b]Department of Chemistry, Hazara University Mansehra, 21300, Khyber Pakhtunkhwa, Pakistan

Corresponding author: Muhammad Rabnawaz

Tel.: +1 517-432-4870;

*E-mail: rabnawaz@msu.edu; Postal address: School of Packaging, Michigan State University, 448 Wilson Road, East Lansing, Michigan 48824-1223, USA.

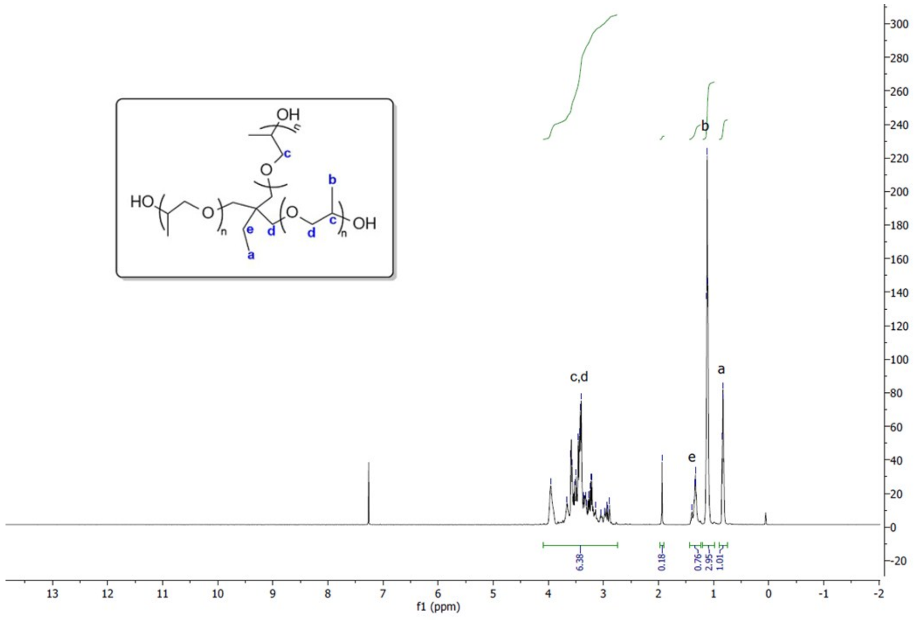

Figure S1. ^{1}H NMR spectrum of the polyol P1 ($CDCl_3$, 500 MHz).

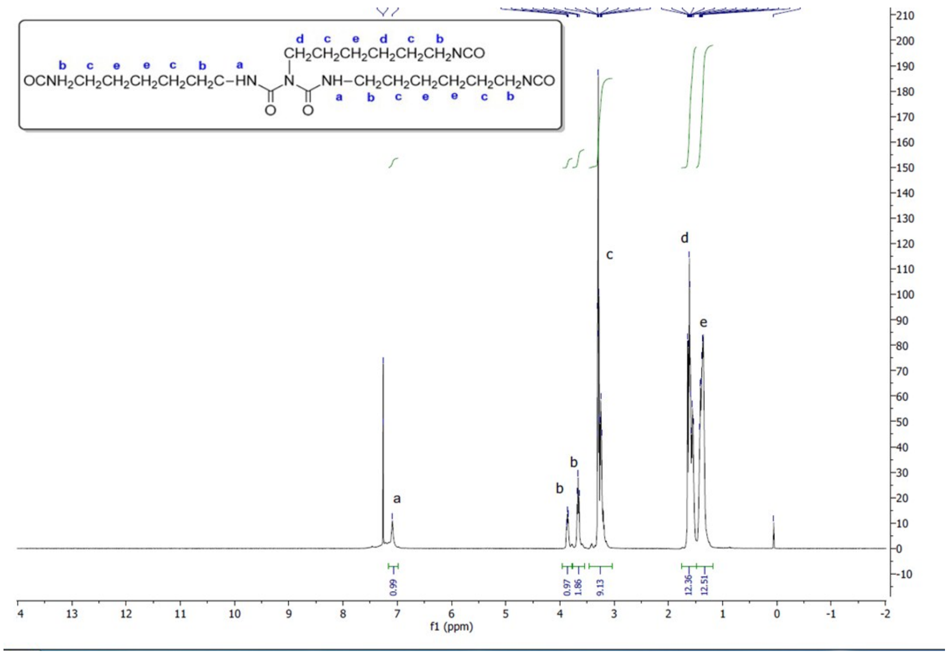

Figure S2. ^{1}H NMR spectrum of hexamethylene diisocyanate trimer (HDIT) ($CDCl_3$, 500 MHz).

2.0 ATR-IR analysis:

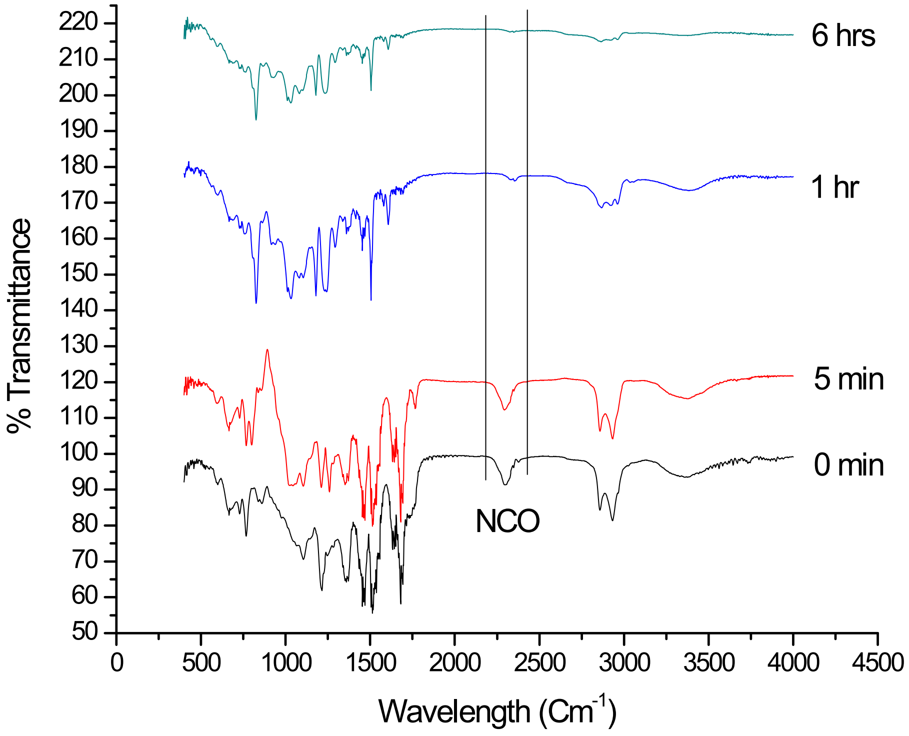

Figure S3. ATR-IR spectra recorded for urethane coating at different intervals of curing treatment at 120 °C.

Vitrification of the bottom layer: PU coatings was applied on a glass slide. These coatings were heated for 0, 1, 2, 5, and 10 min at 120 °C prior to the application of the top layer. Sampels cured for 2 min or less were completely dissolved upon the addition of the top-layer PDSM-NH_2 solution, while those that were cured for 10 min did not allow the PDMS-NH_2 solution to permeate into the coating. While samples with 5 min of thermal treatment not only allowed to permeate PDMS-NH2 solution but also did not dissolve upon the addition of PDMS-NH_2.

Sample compositions:

Table S1. PU thermosets and their composites

Samples		Composition (wt %)
Urethanes	PU1[a]	Polyol:Isocyanate (61.2:38.8)
	PU2	Polyol:Isocyanate:PDMS (61.1:38.6:0.3)
	PU3 [b]	Polyol:Isocyanate:PDMS (61.1:38.6:0.3)
Urethanes/ Nanoclay	PU4 [a]	Polyol:Isocyanate:Nanoclay (60.9:38.8:0.3)
	PU5	Polyol:Isocyanate:Nanoclay: PDMS (60.8:38.7:0.2:0.3)
	PU6[b]	Polyol:Isocyanate:Nanoclay: PDMS (60.8:38.7:0.2:0.3)
Urethanes/ CNC	PU7 [a]	Polyol:Isocyanate:CNC (60.9:38.8:0.3)
	PU8	Polyol:Isocyanate:CNC: PDMS (60.8:38.7:0.2:0.3)
	PU9[b]	Polyol:Isocyanate:CNC: PDMS (60.8:38.7:0.2:0.3)
Urethanes/ GO	PU10[a]	Polyol:Isocyanate:GO (60.9:38.8:0.3)
	PU11	Polyol:Isocyanate:GO:PDMS (60.8:38.7:0.2:0.3)
	PU12[b]	Polyol:Isocyanate:GO:PDMS (60.8:38.7:0.2:0.3)

[a]= No PDMS-NH_2 was used; [b]= prepared via *"in situ"* mixing.

Table S2. The surface roughness of PU samples evaluated using atomic force microscopy (AFM) with contact mode of operation. The root means square (RMS) roughness of the surfaces were evaluated for arbitrary line drown across the edges.

.

3D view of the scanned samples e.g. PU1	*Sample* *RMS roughness (pm)*
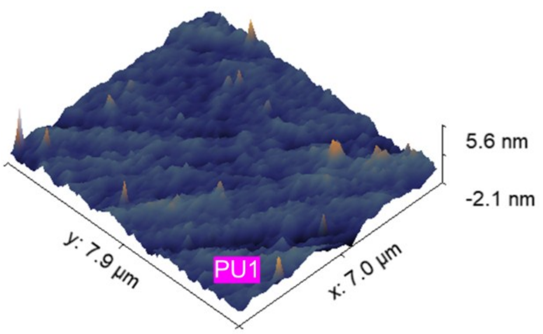	PU1 ---------------------500<br>PU2 ---------------------328<br>PU3 ---------------------890<br>PU5 ---------------------205<br>PU11---------------------190

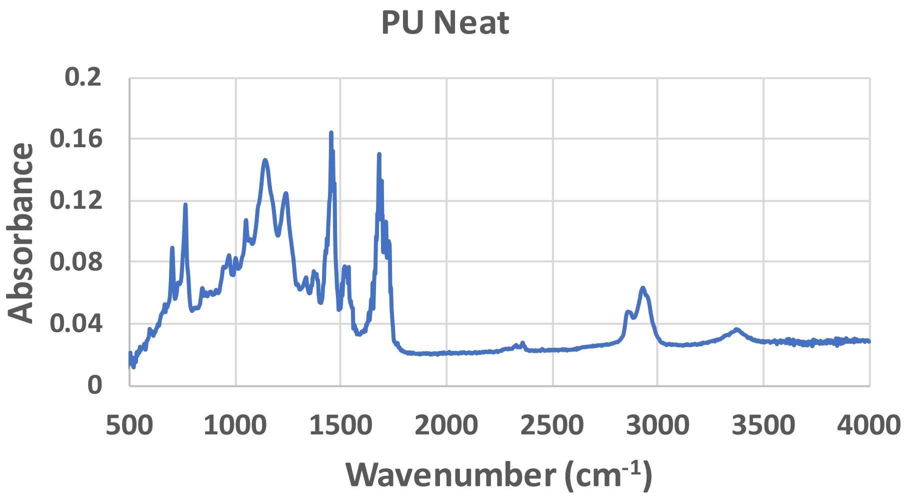

Figure S4. FTIR of fully crosslinked PU after curing at 100 ^{o}C for 4 hrs.

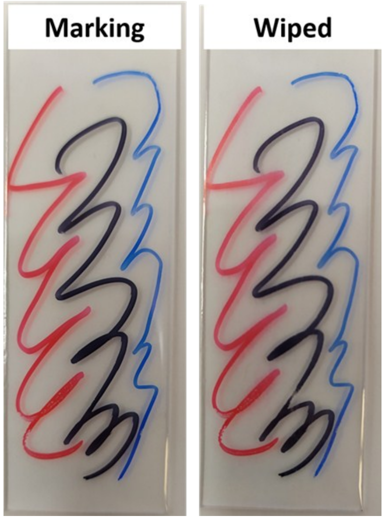

Figure S5 Anti Ink property for fully-cured PU sample treated with PDMS-NH_2 solution (PDMS $_{in}$ hexanes solution (5mg/mL) for 20 seconds. No anti-graffiti properties were witnessed.

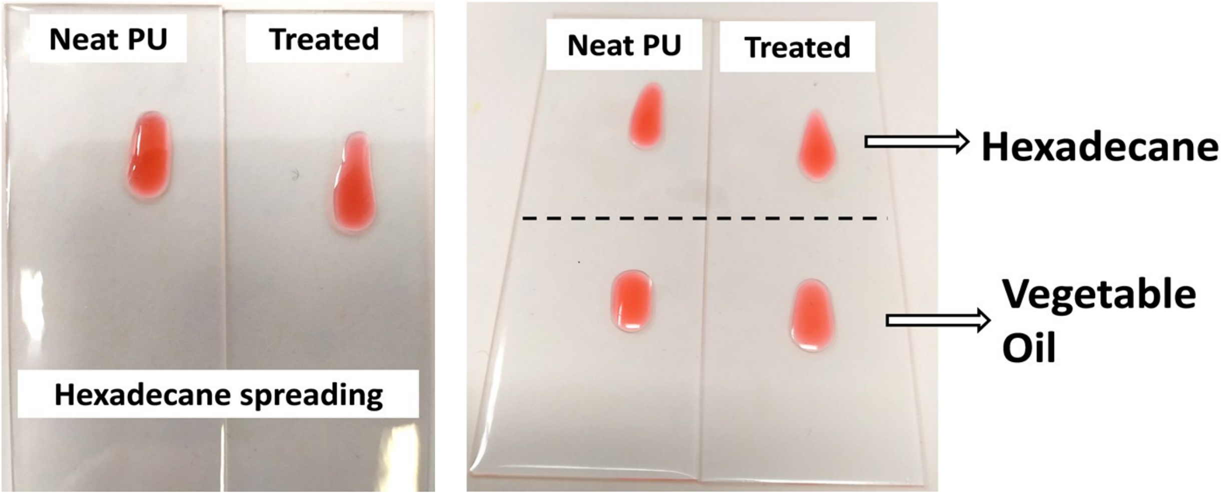

Figure S6 Hexadecane and vegetable oil spread on the surface of fully-cross-linked PU before and after PDMS treatment. No improvement in the repellencies against both polar and non-polar liquids were observed.
